# Density of Langerhans Cells in Nonmelanoma Skin Cancers: A Systematic Review

**DOI:** 10.1155/2020/8745863

**Published:** 2020-04-19

**Authors:** Joanna Pogorzelska-Dyrbuś, Jacek C. Szepietowski

**Affiliations:** ^1^“Estevita” Specialist Medical Practice, Tychy, Poland; ^2^Department of Dermatology, Venereology and Allergology, Wroclaw Medical University, Wroclaw, Poland

## Abstract

Langerhans cells (LCs) are bone marrow-derived dendritic cells (DCs) that represent 2-3% of the entire cell population of the human skin, known to have an ability to present antigens to T lymphocytes. Moreover, there is evidence that LCs are probably capable of inducing the local cytotoxic type T-cell-mediated response against the tumour-associated antigens. In the past two decades, a dramatic increase has been noted in the incidence of basal cell carcinoma (BCC) and squamous cell carcinoma (SCC). The purpose of this study was to critically assess the results of available studies quantitatively assessing the LCs in nonmelanoma skin cancers and try to establish a conclusion of its possible impact on their future treatment. The PubMed, EMBASE, and the Web of Science databases were searched, which returned 948 citations. After a thorough analysis of full article texts, 30 studies have been chosen, including 11 of the BCC, 12 of the SCC specimens, and 7 analysing both tumour types. There was an overall trend towards slightly higher numbers of LCs in BCC than in SCC; however, these tendencies were discrepant between the studies. We presume that such differences could be caused by various staining techniques with a broad spectrum of specificity, including anti-S100, anti-CD1a, and ATPase activity staining used for LCs identification. We hypothesise that as there is a high inconsistency between the results of the studies, as far as the densities of LCs observed in the specimens are concerned, it seems that the mechanism of the influence of LCs on the antitumoural immune response is complicated. Finally, as at present, there is a paucity of available risk scores for the recurrence or progression of BCC or SCC, the creation of classification stratifying that risk including the density of LCs could bring additional information both for the physician and the patient.

## 1. Introduction

Langerhans cells (LCs) are bone marrow-derived dendritic cells (DCs) that represent 2-3% of the entire cell population of the human skin [1]. Their largest number is found in the basal and squamous layers of the epidermis. The LCs are known to have an ability to present antigens to T lymphocytes and migrate from the skin to the regional lymph nodes with the migration increased during the inflammation [2, 3]. They are also probably capable of inducing the local cytotoxic type T-cell-mediated response against the tumour-associated neoantigens expressed by damaged epidermal cells [4].

In the past two decades, a dramatic increase has been noted in the incidence of skin cancers, of which basal cell carcinoma (BCC) and squamous cell carcinoma (SCC) are the most prevalent [5–7]. According to the available data, the incidence of SCC in the European populations is 100.2 in 100.000 men and 72.6 in 100.000 women, while the incidence of BCC is approximately 165 in 100.000 European men and 157 in 100.000 European women [7, 8]. BCC is locally invasive but exhibits very low rates of metastasis, while SCC has metastatic potential and may lead to death [9].

Among many environmental factors, ultraviolet radiation plays a major role in the pathogenesis of human cutaneous malignant transformation, because skin cancers occur most often in skin chronically exposed to the sunlight. Both UVA and UVB can cause free radical activity, mutations in the genes actively controlling cell cycle, or direct DNA damage [10–12].

It appears increasingly evident that the development of skin neoplasms is connected with an impaired response of the immunological system, with a major role played by LCs. However, the relationship between LCs and many other cells of the cutaneous immunological system is still not well understood [3, 13, 14].

Understanding of LC function can help to introduce the new therapeutic methods and may provide new perspectives for the immunosurveillance against skin cancer. Few articles have been published to date raising the question, whether LCs would have a role in the immunosurveillance against skin cancer [13].

Therefore, the purpose of this study was to critically assess the results of available studies quantitatively assessing the LCs in nonmelanoma skin cancers and try to establish a conclusion of its possible impact on their future treatment.

## 2. Methods

Relevant articles quantitatively evaluating the number of LC in BCC and SCC were searched on the 08.07.2019 using PubMed (MEDLINE), EMBASE, and the Web of Science. Relevant articles quantitatively evaluating the number of LC in BCC and SCC were searched using PubMed (MEDLINE), EMBASE, and the Web of Science. The terminology searched for included the following keywords used in various combinations: Langerhans Cells; Squamous Cell Carcinoma, Basal Cell Carcinoma; Skin Cancer; Number; Quantitative assessment. In addition, due to the shortage of recent review articles focused specifically on this field of medicine, the references mentioned in the original papers were searched as well, in order to find their original sources of information. All the titles and abstracts from the search along with the full texts (if necessary) were investigated by two independent reviewers; any discrepancies were clarified by a constructive discussion. We did our best to focus on each individual study included in the systematic review paying attention to selection, performance, detection, and reporting biases. According to the inclusion criteria, the articles must have been written in English without publication time restrictions. Patients must have had a surgically excised nonmelanoma skin cancer (either SCC or BCC) with the number of Langerhans Cells measured using one of the following staining: anti-CD1a antibodies, anti-S100 antibodies, anti-CD207 antibodies, or ATPase activity measurement.

Articles were excluded if the patients had concomitant nonskin cancers undergoing therapy, significant primary or iatrogenic immunological deficiencies, were under the age of 18 years, or the lesions were sampled using the fine-needle aspiration.

Articles not yet published, although available online, were not included. Case reports, conference abstracts, and letters to the editors along with review articles or guidelines were excluded from the analysis. Furthermore, in order to summarise the potential clinical value of continuous assessment of the number of LCs in nonmelanoma skin cancers conducted in the large centres, studies consisting of less than an arbitrary number of five patients were excluded from the analysis. Institutional review board approval was not obtained, as our systematic review involved the retrospective analyses of deidentifying studies that had already been published.

## 3. Results

**Figure 1:**
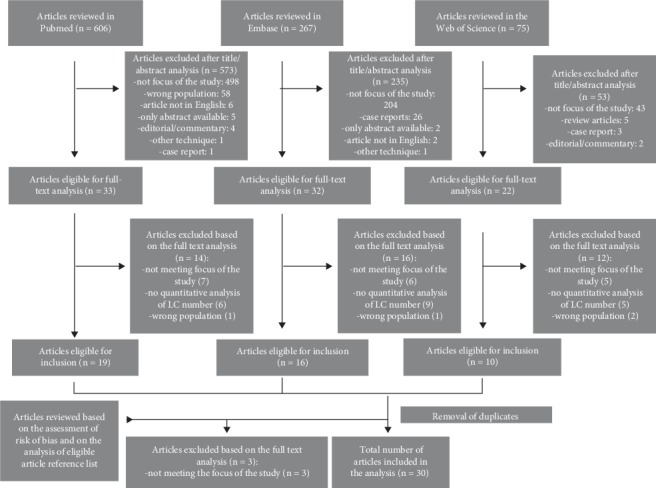
Scheme of selection of the studies.

Searching the literature returned 948 unique citations but due to a relatively limited number of studies investigating the subject, all three sources were analysed independently. Afterwards, they underwent duplicate removal. After a thorough analysis of full article texts, 26 studies have been chosen. Further analysis of the full texts available from the search of all reference sources of relevant articles allowed to include additional four studies finally making up 30 studies retained for the analysis. The scheme of data selection is presented in Figure 1.

There were 11 studies quantitatively analysing the number of Langerhans Cells in Basal Cell Carcinoma in a total of 237 cases, with an average of 22 cases per study. The largest study, published by Bergfelt et al., included 65 cases of BCC [15]. In four studies, ATPase activity was measured, while two utilized anti-S100 antibodies and seven anti-CD1a antibodies. The summary of those studies is presented in Table 1. Twelve studies, presented in Table 2, encompassing 188 cases, analysed the number of LCs in SCC, with the largest conducted in 36 subjects by Wei and Tahan [16]. Out of those, in two anti-S100 antibodies were used, in the other two anti-CD207 antibodies were utilized, while in the remaining nine studies, LCs were stained using anti-CD1a antibodies. Seven studies were conducted on both SCC and BCC specimens, which included 88 cases of SCC and 90 cases of BCC. The largest of those was performed by Shevchuk et al. with 40 cases of each tumour type [17]. They are summarised in Table 3.

### 3.1. BCC

The first study in which the authors tried to assess the number of LCs in BCC was conducted by Azizi et al., who stained the specimens for ATPase activity and compared the density of LCs in the BCC to the perilesional skin [18]. They proved that there was no difference between the two aforementioned sites; however, the study was more concentrated on the morphology of the cells, which was significantly altered in the region of the tumour. Hence, the authors concluded that BCC might arise in areas with the alterations of rather the morphology than the activity of LCs [18]. In the study by Santos et al., antibodies against S-100 were used to identify the LCs [19]. Based on the histological features of the specimens, the authors divided them into two groups: the tumours with lower or higher potential of local aggressiveness. They reported a significant increase in the number of LCs in the normal epidermis when compared to the epidermis superposed to the BCC with lower local aggressiveness. However, there was no significant difference between the number of LCs in the superposed epidermis and the normal epidermis adjacent to the lesion of the BCCs with a high potential of local aggressiveness [19]. The authors stated that a higher number of antigen-presenting cells in the normal epidermis adjacent to the less aggressive tumours could be an indicator of a greater immunological resistance of the epidermis and therefore a limitation of aggressiveness of the neoplasm [19]. On the other hand, it could also be the result of the method of staining, as anti-S-100 antibodies are lacking complete specificity and the increased number of LCs observed in the study could reflect the staining of many other cells.

There is also a number of studies showing no differences in the number of LCs between the benign and malignant cutaneous lesions. With the use of anti-S-100 antibodies, De Mello et al. revealed no difference in the number of LCs in the normal skin and BCC tissue [20]. Azizi et al. compared the density of LCs in BCC to the perilesional skin and proved that there was no difference in the two sites, while Bergfelt et al. compared LCs density in 16 cases of BCC with healthy control and revealed that there was no difference in LCs distribution [18, 21].

Using ATPase method of staining LCs, Alcalay et al. analysed the sensitivity of LCs to simulated solar radiation in patients with BCC and revealed that exposure to radiation resulted in a significant decrease of the number of ATPase-positive LCs [22], while Mardones et al. used anti-CD1a antibody to compare the areas of epidermis overlying and adjacent to the BCC and showed the lower density of LCs in the epidermis overlying the tumour; Rotsztejn et al. demonstrated a decreased number of LCs in BCC using the same staining [23, 24]. In two studies published by Bergfelt et al., respectively, in 1992 and 1993, a reduction in density of LCs in epidermal sheets of BCC was documented with two different techniques. In the first study, two markers for immunohistochemistry: anti-CD1a and ATPase staining; in the second, confocal laser scanning microscopy was used to analyse LCs [15, 25].

### 3.2. SCC

The first, who quantitatively analysed the LCs in squamous cell carcinoma were researchers led by Korenberg, who used an anti-S100 antibody to compare the density of LCs in the inflamed and noninflamed SCC and inflamed and noninflamed keratoacanthoma (KA). The number of LCs was markedly increased in inflamed KA when compared to the other groups, which demonstrated relatively similar expression of LCs. However, a severe limitation of this study is that there was no numerical comparison to the healthy skin [26].

In the study by Wei et al. conducted on thirty-six cases of SCC, the relationship of dendritic cell density in association with tumour grade, mitotic rate, and depth of invasion was analysed. Dendritic cells were identified using S100 immunohistochemistry and their peri- and intratumoural density was determined. The authors proved that the greater peritumoural density of DCs was correlated with a lower rate of metastasis, which suggests the functional involvement of DCs in the immunologic control of SCC. However, the weakness of his study was that the authors also used anti-S100 antibody of an insufficient specificity [16].

Sandvik et al. conducted a quantitative analysis of LCs in the tissue surrounding invasive SCC in immunosuppressed patients and immunocompetent controls using anti-CD 207 antibody [27]. CD207, also called “Langerin,” is a transmembrane receptor present on the surfaces of LCs and a limited number of DCs, which plays an important role in the internalization of antigen to the Birbeck granules specific to LCs, which induces the pathway of antigen preparation and presentation. In the study, the number of LCs in tumour nests was similar in both immunosuppressed and control patients. In comparison with the epithelial tumour nests, the density of LCs in the normal skin was significantly reduced [27]. These results, however, could be caused by changes in the profile of secreted cytokines upon immunosuppression, which fosters the increase in the concentration of inhibiting factors like IL10, which can transform LCs into inactive forms [28].

There are only a few more articles in which decreased numbers of LCs in SCC were proved. Both in analyses of Rotsztejn et al. and Tucci et al., a decreased number of these cells in SCC compared with the control group has been found [29, 30]. On the contrary, Galan et al. compared the number of LCs in SCC and pseudoepitheliomatous hyperplasia of the skin and obtained no differences between both skin pathologies with depletion of LCs in both. Therefore, the authors concluded that decreased LCs were not unique to a malignant process [31].

As the primary role of Langerhans cells in the skin is to present antigens to local, naive T-cells, their role in the process of tumorigenesis has also been studied from the immunological perspective, such as it has been painstakingly performed in the other tumours including those associated with human papillomavirus infection [32, 33].

Takahara et al. tried to correlate the tumour cellular proliferation including stromal fibroblasts, macrophages, and epidermal LCs in the progression of many epidermal tumours including SCC. In the study, higher expression of CD10, a metalloproteinase involved in the degradation of multiple signalling peptides, on fibroblasts was associated with malignant transformation of keratinocytes and reduction of LCs [34]. The results are consistent with the findings derived from the preclinical studies, such as by Margulis et al., who found that the loss of intercellular adhesion, caused mainly by the activity of metalloproteinases, results in the transformation of SCC cells from low to high grade of malignancy [35].

Although there is a high inconsistency between the results of the studies in the densities of LCs observed in the SCC specimens, a general tendency towards their higher number located intratumorally and lower in the epidermis overlying the tumour can be observed.

### 3.3. BCC and SCC

The first who compared the number of LCs in both BCC and SCC were Liebau et al., who compared the density and morphology of LCs using monoclonal anti-CD1a antibodies, however, only in 6 cases of BCC and 9 cases of SCC of the oral mucosa [36]. In BCC, the majority of LCs were located within the surface of the epidermis, and very few within and around tumour cords, while in SCC, an impressive number of positive anti-CD1a cells were found within the tumour, in contrast to the small number of labelled cells within surrounding tissue. In this study, the authors found more CD1a+cells in the more malignant lesions (SCC) [36]. Smolle et al. investigated the intraepithelial density of LCs in skin cancers and detected a significant depletion of LCs in both SCC and BCC, however, with their significantly higher density in SCC [37]. As a matter of fact, one has to note that in comparison with the other articles investigating both types of tumours, the higher density of LCs in SCC was found in three of them: in the aforementioned study by Smolle et al., in the study by Yamaji et al., and in the study by Chen et al.; however, in the last one, the difference between tumour types was very subtle [37–39]. Another goal of the study by Chen et al. was to find a correlation between the LCs density and degree of epithelial differentiation. The level of epithelial differentiation measured by mean nuclear area was negatively correlated with LCs density, but there was no correlation with the immune response in the peritumoural region [37].

Yamaji revealed an increased number of LCs overlying SCC using anti-CD1a and anti-S100 antibodies. However, no similar increase was registered in BCC [38]. For the authors, the degree of LCs increase paralleled the severity of inflammation and inflammatory infiltrates were observed around the tumour nests. The mechanism might be that the LCs are activated and lured to the epidermis by lymphokines secreted by a group of T-cells comprising the dermal infiltrates surrounding tumour cell nests.

## 4. Discussion

Conflicting data on the number of LCs in nonmelanoma skin cancers can be caused by a wide variety of staining techniques used in the studies, including anti-S100, anti-CD1a, and ATPase activity staining. The lower number of LCs in researches using anti-CD1a might be the natural consequence of a fact that this antibody is considered as the most specific marker for LCs. There were approximately 20% excessive cells, when the specimens were stained with ATPase, most probably being the other inflammatory cells. Hence, one can speculate that the results of two studies by Azizi et al. and Alcalay et al., who demonstrated a significantly higher number of LCs than observed in the other studies, could be a consequence of staining for ATPase activity. The presence of S100 protein, including the S100B subtype present on LCs, has already been demonstrated on various skin cells both in physiological and pathological conditions (such as melanocytes, melanoma, or chondroid syringoma); hence, the results of the studies utilizing the more specific anti-CD1a present only on the surface of LCs and other antigen-presenting cells might be the most reliable.

Worth noting is very varying results, as far as both numerical and comparative aspects are concerned. For instance, in the studies by Schreiner et al. and Smolle et al., there was around fourfold difference in the density of LCs per 1 mm^2^, although in opposite favours [37, 40]. On the other hand, the results of the studies by Yin et al. and Chen et al., who applied identical staining methods, indicate the very similar proportion of LCs in either type of tumour when counted per 1.000 keratinocytes [39, 41]. Although, a crucial limitation of all the mentioned studies, which could explain the low reproducibility of the results is the low number of investigated cases in each specific study.

Because conflicting data exist on the number of LCs in skin malignancies, it seems that the mechanism of the influence of LCs on the antitumoural immune response is complicated. Although the discrepancies between the studies can be partly explained by different staining techniques, changes in the local environment in the epidermis overlying tumour could also influence the LCs distribution and density. However, the molecular mechanisms responsible for the function and activity of LCs are still incompletely discovered. It is known that E-cadherin is very important for homing conditions for LCs and adhesion of LCs to laminin and fibronectin is mediated by integrins [25]. However, a direct relationship between LCs and tumour as well as the epithelial growth and keratinization cannot be ruled out [42]. Some authors proposed a regulating mechanism between LCs and keratinocyte differentiation and postulated that this mechanism might be disturbed in nonmelanoma skin cancer [37].

The results of many reports have not always been comparable, because horizontal, as well as vertical sections and various enumeration methods, has been used [43]. Moreover, the different techniques of defining peri- and intratumoural areas were also applied. Hence, it seems difficult to directly compare the results between the studies and draw the conclusions based upon them. However, we observed an overall trend towards slightly higher numbers of LCs in BCC than in SCC.

More recent research suggested a parallel relationship between the expression of E-cadherin—a molecule responsible for adhesion of epithelial cells, already proved to play an important role in the development of cancer metastases—and LC density [30, 44]. However, the true relationship between skin microenvironment (i.e., cytokeratin differentiation) and density of LCs is still unclear and future studies investigating the role of E-cadherin and other markers may be helpful for better understanding of this tumour pathology.

One of the key clinical aspects differentiating SCC from BCC, which has a dramatic prognostic impact, is that the former has a metastatic potential, while the latter has not. In the study by Takahara et al., a significant reduction of LCs in SCC when compared with normal skin was followed by an increased number of stromal macrophages [34]. According to the study by Petersen et al., macrophages infiltrating a tumour secrete multiple matrix metalloproteinases (MMPs) which promote degradation of the extracellular connective tissue [45]. In the presence of lessened intercellular connections, the efflux of LCs from the tumour microenvironment could take place. That could explain why in the tumours of higher local aggressiveness, the number of LCs is significantly lower.

On the other hand, as the primary genetic mutations are rather similar in the course of malignant transformation of both tumours, the difference in their clinical manifestation and prognosis could be caused by the other factors, like the involvement of the immunological system [46].

We are aware of some limitations of our paper. We focused only on Langerhans cells, not searching for more general term as “dendritic cells.” Therefore, some of the relevant studies of the first phases could be omitted. Moreover, we concentrated our search efforts on three databases, including PubMed (MEDLINE), EMBASE, and the Web of Science. We cannot completely exclude the possibility that some studies were not encompassed by the abovementioned databases.

Summarizing, the quantitative assessment of LCs and their correlation with some stromal cells, e.g., in skin cancers, can have prognostic value and be a target for novel antineoplastic therapies. At the moment, there is a paucity of available risk scores for the recurrence or progression of BCC or SCC and the creation of classification stratifying that risk as well as the need for more careful examination could bring additional information both for the physician and the patient. So far, LC density was proposed as a prognostic marker for laryngeal SCCs and colorectal cancer [14, 47]. Moreover, the lack of CD1a expression in the dendritic cells of Barrett's metaplasia may predict its evolution toward oesophageal adenocarcinoma [17]. It is possible that further investigation can contribute not only to the treatment but also to the risk stratification and intensified surveillance and intervention in patients with either squamous or basal skin cancers.

## Figures and Tables

**Table 1:** The summary of the studies investigating the number of LCs in BCC.

First author, date	No of cases	The purpose(s) of the study	Langerhans cells immunostaining measurement method	Measurement location	Cell numbers
Azizi, 1987 [18]	12	Analysis of the number and morphologic features of LC in BCC in sun-exposed sites	ATPase activity staining Calculation in 5-15 fields of vision Then extrapolated per mm^2^	Epidermis overlying the tumours and perilesional skin	IT: 617 ± 214/mm2 PT: 688.5 ± 207/mm2
Alcalay, 1989 [22]	34	Analysis of susceptibility of LC in BCC to modification by UV radiation	ATPase activity staining Calculation in 10-20 fields of vision and extrapolated per mm^2^	Epidermis overlying the tumours	Before radiation: 1011 ± 123/mm2 After radiation: 682 ± 222/mm2
Mozzanica, 1990 [48]	6	Analysis of LCs in BCCs before and after 15 days of local treatment with IFN-a2b	Anti-CD1a, HLA-DR antibodies Calculation in 6 adjacent sections of tumour, extrapolated to 0.25 mm^2^	Intra- and peritumoural	Before treatment IT: 19.6 ± 7.4 PT: 9 ± 5 During treatment: IT: 21.1 ± 6.0 PT: 3.3 ± 1.3
Bergfelt, 1992 [15]	65	Analysis of relationship between exposure to UV and the number of LCs in a BCC and normal skin	ATPase activity and anti-CD1a antibody Calculation per mm^2^ in 5-15 random fields of vision	Intra- and peritumoural	IT: ATPase 506/mm^2^ (mean), range 262-882 CD1a 426/mm^2^ (mean) range 162-677 PT: ATPase 708/mm^2^ (mean) range 450-838 CD1a 626/mm^2^ (mean) range 450-838
Bergfelt, 1993 [21]	16	Analysis of influence of chronic sun/PUVA exposure on the number of LC and tumour development	Anti-CD1a antibody and ATPase staining Calculation in 5-15 random fields then extrapolated to per 1 mm^2^	Epidermis overlying the tumours	Hand: ATPase: 434 ± 67/mm2 CD1a: 371 ± 104/mm2 Buttock: ATPase: 644 ± 50/mm2 CD1a: 511 ± 83/mm2
Bergfelt, 1994 [25]	15	Comparison of LC quantification with light microscopy and in vitro confocal microscopy	Anti-CD1a antibodies/CM Calculation horizontally and vertically by per mm^2^ or unit length (0.2 mm)	Interfollicular part of epidermis overlying the tumours	Horizontally: 412 ± 113/mm2 Vertically: 4.76 ± 1.74/unit length
De Melo Jr, 2006 [20]	35	Computerized quantitative analysis of LCs in the cutaneous tumours	Anti-S100 antibody Calculation in 3 sites of the tumour and extrapolated to area per field (12,234 um^2^)	Epidermis overlying the tumours	19.75 ± 5.81/area per field
Rotsztejn, 2009 [23]	20	Analysis of LC number in BCC in the sun-exposed skin	Anti-CD1a antibody Calculation per 10 HPFs	Intra- and peritumoural	IT: 0.35 ± 0.88 (range 0.0-3.0) PT: 8.30 ± 4.23 (range 3.0-18.0)
Mardones, 2009 [24]	12	Comparison of density and morphology of LCs in the epidermis overlying and surrounding BCC	Anti-CD1a antibody Calculation per 1 *μ*m^2^	Epidermis overlying the tumour and max 2500 *μ*m from its border	IT: 12.99 ± 5.61 PT: 23.27 ± 8.67
Santos, 2010 [19]	14	Quantification of LC on the epidermis of BCC depending on the local aggressiveness	S100 antibody Calculation in 20 FCUs per 1 mm^2^	Intra- and peritumoural	Low aggressiveness: IT: 4.61 ± 2.44, PT: 6.70 ± 2.68 High aggressiveness: IT: 5.21 ± 2.74, PT: 5.05 ± 2.38
Evangelou, 2012 [49]	8	Analysis of influence of PDT on the number of LC in superficial BCC	Anti-CD1a antibody Calculation per one HPF	Epidermis overlying the tumours+skin from site distal to the tumour	Before PDT: 6.5 ± 0.6 After 1 hour post-PDT: 2.6 ± 0.8 After 24 hours post-PDT: 1.5 ± 0.6

BC: basal cells; BCC: basal cell carcinoma; CM: confocal microscopy; FCU: fundamental counting unit; HPF: high-power fields; IFN-a2b: interferon alfa 2b; IT: intratumoural; KC: keratinocytes; PDT: photodynamic therapy; PT: peritumoural; PUVA: psoralen and ultraviolet A; UV: Ultraviolet.

**Table 2:** The summary of the studies in which the number of LCs was measured in SCC.

First author, date	No of cases	The purpose(s) of the study	Langerhans cells immunostaining measurement method	Measurement location	Cell numbers
Korenberg, 1987 [26]	12	Quantification of LCs in inflamed and noninflamed keratoakanthoma and SCC	Anti-S100 antibody Calculation in 4 HPFs	Intratumoural	Inflamed SCC: 2.6 (range 0-8) or 3.2 (0-10) Noninflamed SCC: 2.0 (0-8) or 3.0 (0-7)
Tucci, 1998, [29]	5	Analysis of relationship between transformation of keratinocytes and markers of oncogenesis	Anti-CD1a antibody Calculation per 0.01 mm^2^ in 10 fields of vision	Intratumoural	2.98 ± 1.94
Wei, 1998 [16]	36	Analysis of association of S100+ cells presence in SCC and presence of metastates	Polyclonal anti-S100 antibody Three cases with/with no metastasis stained with anti-CD1a antibody Calculation per mm^2^	Intra- and peritumoural	PT: mean 314 ± 50/mm2 (range: 0-1243) When stained with anti-CD1a 57% of S100+ cells were identified IT: mean 317 ± 42/mm2 (range: 0-893) No diffrences when stained with CD1a
Berhane, 2001 [50]	19	Analysis of progression of AK to SSC	Anti-CD1 antibodies Calculation by the number per mm^2^	Intratumoural	CD1: 277 ± 77 cells/mm2
Ko, 2006 [51]	10	Comparison of BCL-2 and CD1a staining in various skin pathologies	Anti-CD1a antibody Calculation in 2 HPFs of 0.25 mm^2^	Intratumoural	15.9 ± 12.2
Rotsztejn, 2006 [30]	5	Analysis of LC number in vulvar SCC	Anti-CD1a antibody Calculation per 10 HPFs	Intratumoural	1.0-1.7 (range 0.0-4.0)
Galan, 2007 [31]	12	Comparison of LC in PEH vs SCC	Anti-CD1a antibodies Calculation in two different 0.5 mm^2^ fields of vision	Epidermis overlying the tumours	7.5/0.5 mm^2^
Rotsztejn, 2007 [52]	13	Analysis of LC number in vulvar SCC	Anti-CD1a antibody Calculation per 10 HPFs	Intratumoural	0.85±−0.90 (range 0.0-2.0)
Bluth, 2009 [52]	10	Analysis of immune microenvironment and function of tumour myeloid DCs	Anti-CD1a and anti-CD207 antibodies Calculation based on computer-assisted manual counting of positive cells Extrapolation to unit area (100,000 *μ*m^2^)	Intra- and peritumoural	IT: CD1a: 7.1/100,000 *μ*m^2,^ (median) CD207: 7.0/100,000 *μ*m^2^ (median) PT: CD1a: 0.0/100,000 *μ*m^2,^ (median) CD207: 0.0100,000 *μ*m^2^ (median)
Takahara, 2009 [34]	15	Analysis of correlation between tumour cell proliferation and epidermal LC	Anti-CD1a antibody Calculation per three HPFs	Intratumoural	9.4 ± 2.77
Sandvik, 2014 [27]	30 (15 RTR, 15 control)	Quantification of cells in surrounding of SCC and LC in epithelial tumour nests Comparison with IC patients	Anti-CD207 antibody Calculation among the keratinocytes in the tumour in average 8 random places Then extrapolated to number per mm^2^	Intra- and peritumoural	Intratumoural: -IS patients: median 30/mm^2^ -IC patients: median 35/mm^2^
Gomes, 2015 [53]	21	Comparison of density and distribution in epithelium and IDC of LC in AC and SCC	Anti-CD1a antibody Calculation in seven fields of vision Then extrapolated to 1 mm^2^	Epithelium and connective tissue of the tumour	Epithelium: 44.44 ± 20.65 Connective tissue (interstitial dendritic cells): 21.16 ± 12.48

AC: actinic cheilitis; AK: actinic keratosis; CD: cluster of differentiation; DC: dendritic cells; HPF: high-power fields; IC: immunocompetent; IS: immunosuppressive; IT: intratumoural; LC: Langerhans cells; PEH: pseudoepitheliomatous hyperplasia; PT: peritumoural; RTR: renal transplant recipient; SCC: squamous cell carcinoma.

**Table 3:** The summary of the studies in which the number of LCs was measured in BCC and SCC.

First author, date	No of cases (SCC/BCC/)	The purpose(s) of the study	Langerhans cells immunostaining measurement method	Measurement location	Cell numbers
Yin, 2012 [41]	10/10	Analysis of difference in count of CD1a+ and HLA-DR+ cells in different tumours of skin associated with solar radiation	Anti-CD1a and HLA-DR antibody calculation per 1000 keratinocytes in five different fileds of view of the specimen	NA	BCC: 38.47 ± 3.10 per 1000 KC SCC: 38.38 ± 4.05 per 1000 KC
Yamaji, 1987 [38]	9/6	Analysis of the number of LC in skin tumours	Anti-CD1a and anti-S100ß antibody Calculation per 100 basal cells in three random fields of each specimen	Epidermis overlying the tumours	SCC: 34 ± 3 per 100 BC for CD1a+ and 28 ± 4 for S-100ß+ BCC: 25 ± 6 per 100 BC for CD1a+ and 24 ± 6 for S-100ß+
Schreiner, 1995 [40]	10/13	Analysis of LC number in skin cancers	Anti-CD1a and CD4 antibodies Calculation in 10 randomly selected areas per 1 mm^2^	Epidermis overlying the tumours	SCC: mean 47/mm^2^ BCC: mean 197/mm^2^
Liebau, 1986 [36]	9∗/6	Comparison of density and morphology of LCs in the head and neck skin tumours	Anti-CD1, HLA-DR antibody Calculation in 20 HPFs in each analysed tissue layer as percentage of total number	Epithelium, basement membrane zone and connective tissue	Epithelium: 16% in SCC and 21% in BCC Connective tissue: 9% in SCC and 4% in BCC
Chen, 1988 [39]	4/10	Analysis of class II antigen expression in cutaneous tumours	Anti-CD1a, anti-HLA-DR, anti-HLA-DQ and anti-OKIa1 antibodies Calculation per 1,000 keratinocytes or as percentage of mononuclear cells	Intra- and peritumoural	BCC: IT: 4 ± 3/1,000 KC PT skin: 38 ± 13/1,000 KC (when stained with CD1a). Different stains: OKIa1: 28 ± 15/1,000 KC; HLA-DR: 27 ± 10/1,000 KC; HLA-DQ: 8 ± 8/1,000 KC SCC: PT: 40 ± 26/1,000 KC
Shevchuk 2014 [17]	40/40	Comparison of LC quantification assessing either CD1A or CD207	Anti-CD1a and anti-CD207 antibodies Calculation of CD1A and CD207 cells per total 1000 cells	Intratumoural	SCC: CD1a: 1.0% ± 1.0 per 1000 cells CD207: 0.5% ± 0.5 per 100 cells BCC: CD1a: 2.2% ± 1.0 per 1000 cells CD207: 0.8% ± 0.6 per 1000 cells
Smolle, 1986 [37]	6/5	Analysis of correlation between number of tumour intraepithelial LC, periumoural infiltrate and epithelial differentiation	Anti-CD1a antibody Calculation in 5 consecutive fields and extrapolated to per 1 mm^2^	Intra- and peritumoural	SCC: 100 ± 21/mm2 (range 9-242) BCC: 28 ± 6/mm2 (range: 5-79)

BCC: basal cell carcinoma; CD: cluster of differentiation; HLA: human leukocyte antigen; HPF: high-power fields; IT: intratumoural; KC: keratinocyte; PT: peritumoural; SCC: squamous cell carcinoma. ∗oral squamous cell carcinoma.
